# Increase of GII.17 Norovirus Infections During the 2024–2025 Season in Multiple Cities, China

**DOI:** 10.1002/jmv.70858

**Published:** 2026-03-02

**Authors:** Wenjing Zheng, Xinyue Mu, Shaoyan Wang, Tianyi Qiu, Xuanyi Wang

**Affiliations:** ^1^ Institutes of Biomedical Sciences Fudan University Shanghai China; ^2^ Shanghai Institute of Infectious Disease and Biosecurity, Key Laboratory of Medical Molecular Virology of MoE&MoH, Shanghai Medical College Fudan University Shanghai China; ^3^ Intelligent Medical Institute Fudan University Shanghai China; ^4^ Institute of Clinical Science, Clinical Center of Biotherapy, Zhongshan Hospital, Shanghai Institute of Infectious Disease and Biosecurity, Intelligent Medicine Institute, Shanghai Medical College Fudan University Shanghai China

**Keywords:** China, genotyping, GII.17[P17] stains, molecular epidemiology, norovirus, phylogenetic analysis

## Abstract

A significant numerical increase of genotype GII.17 norovirus‐associated sporadic infections was observed in multiple cities in China during the 2024–2025 season. We collected fecal samples from hospitalized children with acute gastroenteritis from 29 sentinel hospitals nationwide to screen for norovirus. A total of 18.7% (*n* = 412) of the samples tested positive, with a decrease in genotype GII.4 (27% of all positive cases) coinciding with an increase in GII.17 (62%). Comparative phylogenetic analysis of RNA‐dependent RNA polymerase and capsid gene sequences revealed that these GII.17[P17] strains were most closely related to strains circulating in the United States and European countries during 2023–2024, with which they shared a recent common ancestor. The evolving epidemiology of norovirus, characterized by the circulation of multiple genotypes, highlights the need for ongoing surveillance and research to better understand its impact and guide the development of vaccines.

## 1 Introduction

Norovirus is a non‐enveloped, single‐stranded RNA virus belonging to the *Caliciviridae* family and characterized by considerable genetic diversity, with multiple genogroups and genotypes circulating globally [1]. Each year, about 136 000–278 000 deaths occur due to norovirus acute gastroenteritis, and the vast majority are children under the age of 5 [1, 2, 3]. Norovirus RNA genome possesses three open reading frames (ORF1, ORF2, and ORF3), encoding non‐structural proteins, major capsid protein VP1, and minor capsid protein VP2, respectively [4]. VP1 is the most critical component, of which the hypervariable P2 subdomain contains antigen‐presenting sites and carbohydrate‐receptor binding regions, directly interacting with histo‐blood group antigens (HBGAs) and being the primary target of neutralizing antibodies [5, 6]. Due to immune selection pressure, the virus's long‐term evolutionary dynamics are primarily shaped by antigenic drift driven by point mutations, especially the P2 subdomain, which exhibits a high rate of amino acid substitutions [7, 8]. Based on the complete VP1 amino acid sequence, at least 10 genogroups (GI–GX) are divided, and GI, GII, GIV, GIX (formerly GII.15), and GVIII are known to infect humans [9]. Typical GII.4 genotype variants have emerged every 2–4 years, accounting for over 95% of cases worldwide [10, 11]. Besides, recombination with non‐structural protein RNA‐dependent RNA polymerase (RdRp) of different types also promotes the norovirus evolution.

Over the past two decades, epidemic norovirus genotype in China has generally shown a GII.4‐dominated pattern, occasionally displaced by emerging variants or recombinants. From the mid‐2000s until around 2012, GII.4 variants, such as Yerseke_2006a, Den Haag_2006b, and New Orleans_2009, were cycling alternately [12, 13]. Then the GII.4 Sydney_2012 variant widespread replacement of earlier GII.4 lineages occurred worldwide [14]. Non‐GII.4 genotypes briefly supplanted GII.4, such as GII.17 that emerged around 2014–2015 [15, 16, 17], and GII.2 that caused outbreaks in 2016–2017 [18, 19]. Thereafter, multiple genotypes were co‐circulating, due to the P16 polymerase type increasingly recombining with GII.4 Sydney, GII.3, and other capsid types [20, 21, 22], and surveillance in certain regions, such as Beijing, has indicated a rising proportion of non‐GII.4 genotypes [23]. However, no vaccine for norovirus is currently available. Only two of the most advanced candidates in development are bivalent vaccines targeting the genotypes GI.1 and GII.4 [24].

Considering the ongoing antigenic drift and recombination events of norovirus, as well as the challenges in vaccine development, underscores the necessity for epidemiological surveillance to guide prevention and control. Our epidemiological study was conducted in multiple cities in China, among children under 5 years of age who were hospitalized for acute gastroenteritis, during 2024–2025. The aims were to (i) characterize the latest norovirus epidemiological patterns, (ii) identify novel or rare antigenically distinct strains in time, and (iii) provide evidence to support policymakers in guiding vaccine development and updating prevention policies. Notably, our results revealed a significant increase of GII.17 norovirus infection in China this season. Similar reports from many European countries and the United States during 2023–2024 [25, 26, 27]. A phylogenetic analysis of the GII.17 virus sequences in this study was conducted to trace the probable sources of virus transmission, providing valuable insights into evolutionary dynamics and informing intervention priorities.

## 2 Materials and Methods

### 2.1 Participants and Specimens Collection

**FIGURE 1:**
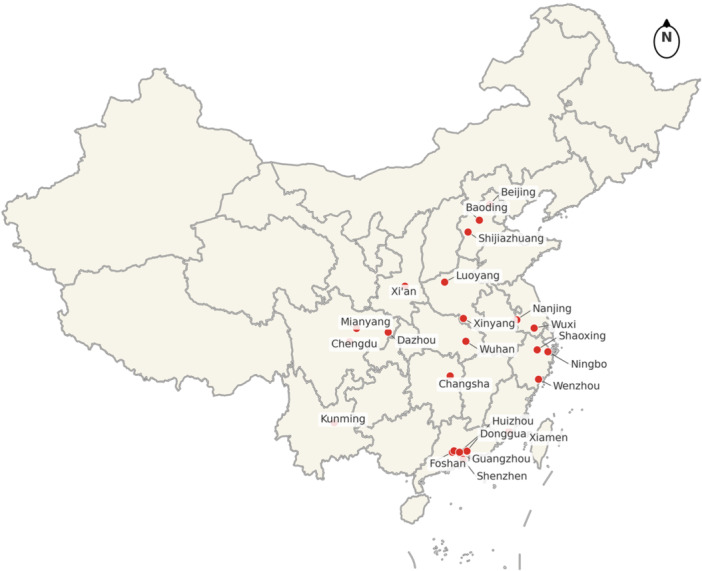
Map of 23 cities where samples were collected in a densely populated region of China. Among these cities, there are three sentinel hospitals in Chengdu, two each in Wenzhou, Changsha, Shenzhen, and Xiamen, and one each in the other cities. In total, there are 29 sentinel hospitals.

Between October 2024 and April 2025, fecal samples were collected from children under 5 years of age who were hospitalized for acute gastroenteritis (AGE): (i) Three or more episodes of watery or loose stools within 24 h and/or severe vomiting, (ii) Visited a sentinel hospital, or received intravenous rehydration treatment for 2 or more consecutive days in an outpatient setting for AGE. The samples were collected from 29 children's hospitals/pediatric departments located in 23 cities across China's densely populated provinces (Figure 1). Laboratory testing was performed at the Key Laboratory of Medical Molecular Virology, Fudan University, and the samples were stored at −30°C.

### 2.2 Ethics Approval and Consent to Participate

This study was reviewed and approved by the Institutional Review Board (IRB) of the Institutes of Biomedical Sciences, Fudan University (Approval No. 2020‐001), and was registered in the Chinese Clinical Trial Registry (Registration No. CTR20201866). Fecal sample collection was conducted as part of routine diagnostic testing for children with diarrhea in hospitals. All clinical samples were de‐identified and analyzed anonymously. No personally identifiable information was involved in this study. Therefore, the requirement for written informed consent was waived by the IRB. All procedures were performed in accordance with the ethical guidelines of the Fudan University Ethics Committee.

### 2.3 Norovirus Detection, RNA Extraction, and RdRp–VP1 RT‑PCR

Fecal suspensions (10%) were prepared in 1× PBS and thoroughly vortexed to mix the viruses, then processed by centrifugation. Viral RNA was extracted from 0.2 mL fecal suspensions using the TIANLONG DNA/RNA Extraction kit (TianLong Science & Technology, China). The RT‐PCR was performed using a QIAGEN One Step RT‐PCR kit (QIAGEN, Germantown, MD, USA). To determine the norovirus genotypes, an approximately 560‐nucleotide region of the viral genome, spanning the ORF1/ORF2 junction, was amplified. This region encodes the C‐terminal portion of the RNA‐dependent RNA polymerase (RdRp) and the N‐terminal portion of the S domain of the capsid protein VP1 [28]. Primer pairs MON431/G2SKR (for GII) and MON432/G1SKR (for GI) were used [29]. All purified PCR products were subjected to sequencing at the Tsingke Biotechnology Co. Ltd., China. Genotyping was determined using online norovirus typing tools (https://www.rivm.nl/mpf/typingtool/norovirus/) that analyze the VP1 (capsid) protein for G‐typing and the nucleotide sequences of the RdRp region for P‐typing.

### 2.4 Sequence Filter

Screened high‐quality sequences from all the sequencing data of the GII.17 virus and included them in phylogenetic analysis. Using the Phred quality score [30, 31], a standardized metric for assessing base‐calling accuracy in DNA sequencing, we set a quality control that sequences were retained only if their average Phred score was ≥ 30 and the length of bases with Q scores ≥ 20 (Q20) was ≥ 500 bp. To ensure representation, at least one sequence should be retained for each city.

### 2.5 Phylogenetic Analysis

To trace the main circulating GII.17 strains, phylogenetic analyses were performed on the genomic regions amplified above, along with a nucleotide and amino acid identity analysis between the GII.17[P17] strains detected in this study and other globally relevant strains available in GenBank (Supplementary File 1), corresponding to distinct variants from different periods: (i) early 2000s (France, 2002), (ii) mid‐2000s (USA, 2005), (iii) mid‐2010s (Japan, 2014), (iv) variants with amino acid insertion mutations emerging in the mid‐2010s (Japan, 2015), and (v) sequences from epidemic and sporadic cases reported in multiple countries (2021–2025). Sequence alignment and trimming were performed using MEGA X [32]. The base substitution model (GTR + Γ) was selected using the model selection tool within MEGA X. A maximum likelihood (ML) phylogenetic tree was constructed under this model, with branch support assessed using the ultra‐fast bootstrap method (UFBoot; 10 000 replicates). The tree was visualized using ChiPlot [33]. Pairwise nucleotide distances (p‐distance) were calculated between sequences. To validate the ML topology, a phylogenetic tree was also constructed using the Neighbor‐Joining (NJ) method. Recombination analysis was performed using SimPlot software.

### 2.6 Statistical Analysis

Statistical analyses were performed using IBM SPSS Statistics 20.0 and R version 4.5.1. Data were presented as frequency and percentage. Categorical variables were compared using the Chi‐square test, and a *p* value of less than 0.05 was considered statistically significant.

## 3 Results

### 3.1 Characteristics of Norovirus Epidemiological Patterns in China During the 2024–2025 Season

**FIGURE 2:**
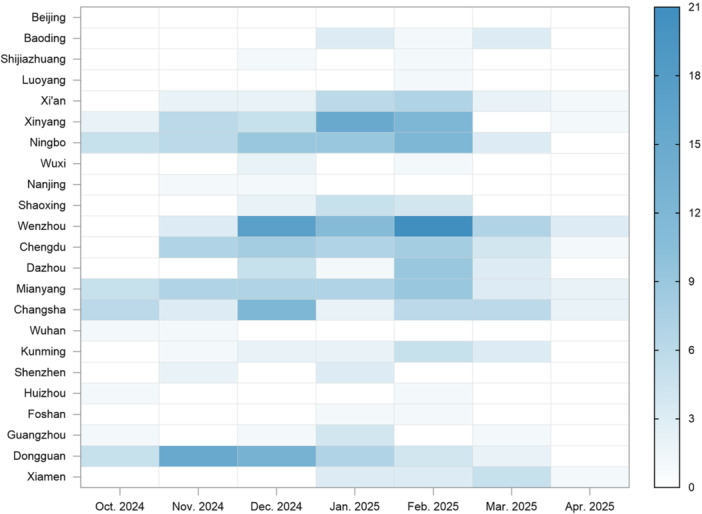
Heatmap of norovirus‐diarrhea cases detected monthly in 23 cities in China.

From October 2024 to April 2025, a total of 2200 fecal samples were collected in 23 cities across China, of which 412 (18.7%) tested positive for norovirus. The monthly number of positive cases varied among these cities (Figure 2). Temporally, norovirus infection cases have been rising month by month, peaking in February and then declining rapidly, indicating a clear late‐winter maximum (*p* < 0.001). Spatially, the infection intensity varies significantly among different cities (*p* = 0.014). A small set of cities accounted for a disproportionate share of positives over the period: Wenzhou (62 cases), Dongguan (46), Ningbo (44), Xinyang (41), Mianyang (40), Changsha (37), and Chengdu (35) led cumulative counts. Monthly dominance shifted modestly: Dongguan led in November (15, 27.3% of that month's total cases), Wenzhou led in December (17, 20.2%) and February (21, 19.8%), and Xinyang led in January (15, 17.4%). Wenzhou also remained the largest single‐city contributor in March (7, 16.7%) and April (3, 27.3%). Consistency differed by location. Changsha and Mianyang showed non‐zero positives in all 7 months, indicating norovirus persistent low‐to‐moderate activity. In contrast, several northern and Pearl River Delta cities exhibited intermittent or near‐zero activity (e.g., Beijing and Shijiazhuang consistently detected near‐zero positives).

### 3.2 Re‐Emergence of GII.17 Strains in China During the 2024–2025 Season

Genotyping analysis revealed that GII.17[P17] was the predominant strain, accounting for 62% (255/412) of all positive cases. Other detected genotypes followed as GII.4[P16] at 26%, GII.3[P12] at 10%, GII.4[P31] at 1%, and GII.2[P16], GII.8[P8], and GI.3[P13] together at 1% (Figure 3a). In 73% of cities, GII.17 was the predominant norovirus genotype detected in positive cases, whereas the distribution of GII.4 strains was observed to be slightly higher than that of GII.17 strains in Luoyang, Wuxi, Kunming, Shenzhen, Guangzhou, and Dongguan (Figure 3b). Almost all the positive cases belonged to the GII genogroup. Only one case caused by the GI genogroup was detected in Dazhou. Additionally, two relatively rare genotypes, GII.8 and GII.2, were detected in Ningbo. In the GII.4 genotype, the predominant strain was GII.4[P16] recombination. Only three cases caused by the GII.4[P31] strain were detected in Wenzhou. GII.17 genotype was all compatible with the P17 type of polymerase, and no other recombinant types were detected. Trends of different genotypes detected over time (Figure 3c) highlight the pronounced fluctuation of GII.17 strain and its lower activity in April.

FIGURE 3The genotype‐specific characteristics of norovirus infection in China. (a) Overall distribution of norovirus genotypes in a total of 412 positive samples. (b) Distribution of GII norovirus samples typed as genotype GII.17 or GII.4 by cities (except for Beijing, as no positive cases were detected). (c) The monthly detection quantities of the main genotypes of norovirus. (d) Number of different norovirus genotype cases by age groups.

**Figure:**
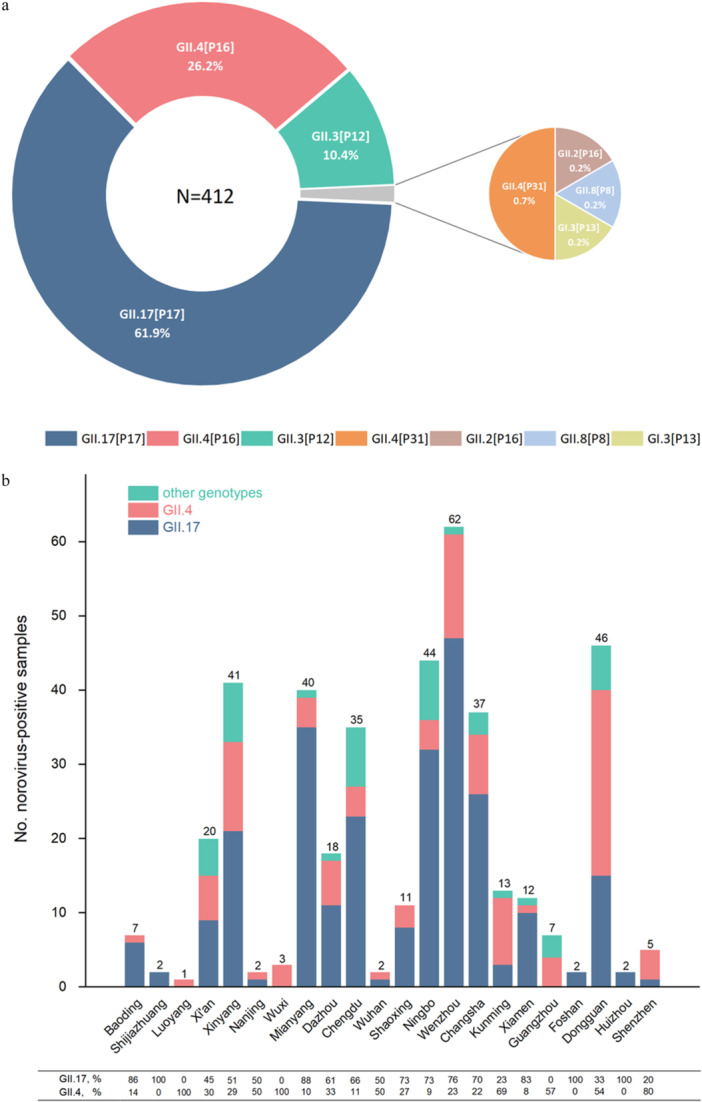

**Figure:**
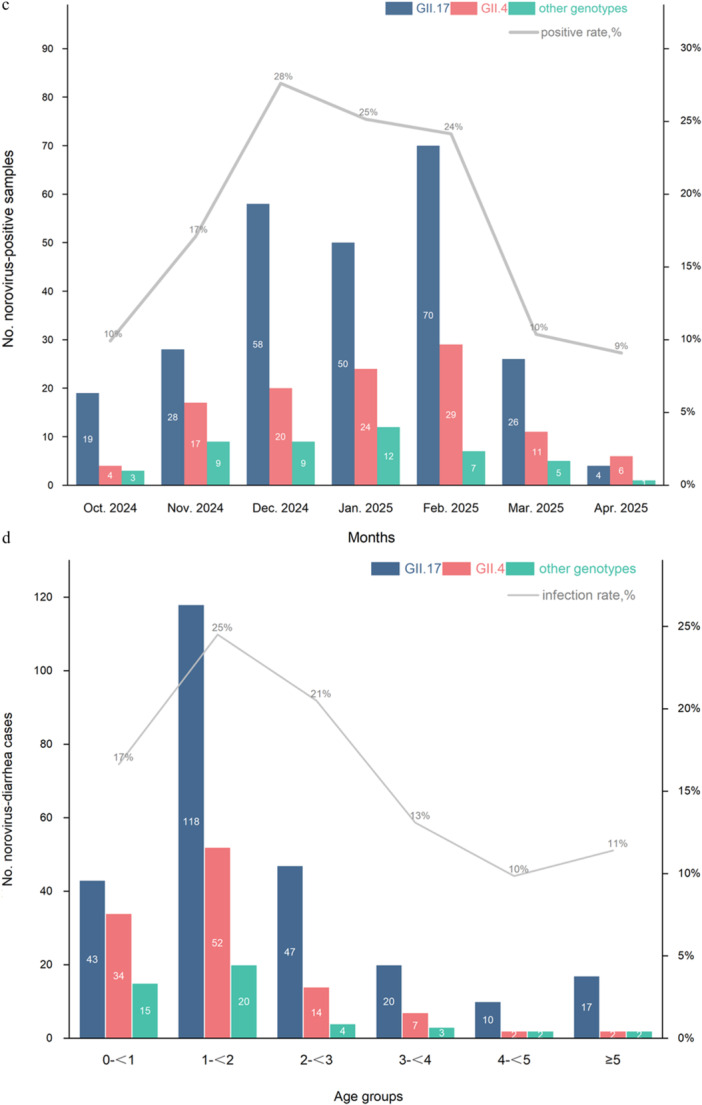

The fecal samples were obtained from hospitalized children diagnosed with AGE, aged 0–6 years. Norovirus infection detected varied significantly across different age groups (*p* < 0.001). Notably, the 1– < 2 years age group exhibited the highest infection rate, reaching a peak of 24.5% (95% CI: 21.5%–27.5%), with 118 cases for GII.17, 52 for GII.4, 19 for GII.3, and 1 for GII.8 (Figure 3d). The GII.17 virus was the predominant cause across all age groups. As age increased, the total number of norovirus infections decreased, with the 4– < 5 years age group showing the lowest cases (10 for GII.17, 2 for GII.4, 1 for GII.3, and 1 for GI.3).

### 3.3 Risk Assessment for Norovirus Infection

Multivariable logistic regression analysis was conducted to examine various factors influencing norovirus infection, focusing on sex, age, season, and location. Since including 22 cities directly was not feasible, we grouped them into four geographical locations (North, East, Midst, and South areas). The seasons were defined as Autumn (October–November), Winter (December–February), and Spring (March–April) (Table 1). The male‐to‐female ratio is approximately 1.5:1 with no significant difference (*p* = 0.467), and the odds of infection are similar for both males and females. The 1– < 2 years age group shows a significantly higher odds of norovirus infection compared to the 0– < 1 years group, with an OR of 1.65 (95% CI: 1.25–2.20) and a *p*‐value of 0.001, indicating a notable risk factor. While other age groups do not show significant differences, the odds of infection in children aged 2– < 3 years are also noteworthy.

**TABLE 1:** Logistic regression analysis of risk factors for norovirus infection.

Variable	The number of patients [*n* (%)]			OR (95% CI)	*p*‐value
	Total	NV positives	NV negative		
Sex					
Female	885 (40.2)	159 (18.0)	726 (82.0)	ref.	—
Male	1315 (59.8)	253 (19.2)	1062 (80.8)	1.09 (0.87–1.37)	0.467
Age					
0– < 1	553 (25.1)	92 (16.6)	461 (83.4)	ref.	—
1– < 2	775 (35.2)	190 (24.5)	585 (75.5)	1.65 (1.25–2.20)	0.001
2– < 3	317 (14.4)	65 (20.5)	252 (79.5)	1.26 (0.88–1.80)	0.211
3– < 4	229 (10.4)	30 (13.1)	199 (86.9)	0.77 (0.48–1.20)	0.254
4– < 5	142 (6.5)	14 (9.9)	128 (90.1)	0.56 (0.30–1.00)	0.062
5– < 6	117 (5.3)	12 (10.3)	105 (89.7)	0.59 (0.29–1.08)	0.106
≥ 6	67 (3.0)	9 (13.4)	58 (86.6)	0.79 (0.35–1.60)	0.532
Season^a^					
Autumn	578 (26.3)	80 (13.8)	498 (86.2)	ref.	—
Winter	1096 (49.8)	279 (25.5)	817 (74.5)	2.22 (1.69–2.95)	< 0.001
Spring	526 (23.9)	53 (10.1)	473 (89.9)	0.75 (0.52–1.09)	0.139
Location^b^					
North	460 (20.9)	71 (15.4)	389 (84.6)	ref.	—
East	608 (27.6)	122 (20.1)	486 (79.9)	1.52 (1.09–2.12)	0.013
Midst	715 (32.5)	145 (20.3)	570 (79.7)	1.48 (1.08–2.04)	0.016
South	417 (19.0)	74 (17.7)	343 (82.3)	1.29 (0.89–1.85)	0.178

^a^ The seasons were defined as Autumn (October–November), Winter (December–February), and Spring (March–April).

^b^ The locations were divided into North (Baoding, Shijiazhuang, Luoyang, Xi'an, and Xinyang), East (Ningbo, Wuxi, Nanjing, Shaoxing, and Wenzhou), Midst (Chengdu, Dazhou, Mianyang, Changsha, Wuhan, and Kunming), and South (Shenzhen, Huizhou, Foshan, Guangzhou, Dongguan, and Xiamen) areas.

Moreover, winter is associated with significantly higher odds of norovirus infection (OR = 2.22, 95% CI: 1.69–2.95, *p* < 0.001), indicating a clear seasonal peak in winter months. The Eastern and Central regions of China are significantly associated with higher odds of norovirus infection compared to the Northern region, with odds ratios of 1.52 (95% CI: 1.09–2.12, *p* = 0.013) and 1.48 (95% CI: 1.08–2.04, *p* = 0.016), respectively. In contrast, the Southern region does not show a statistically significant difference in the odds of infection relative to the North (OR = 1.29, 95% CI: 0.89–1.85, *p* = 0.178).

### 3.4 Genetic and Evolutionary Characteristics of Emergent GII.17 Strains

**FIGURE 4:**
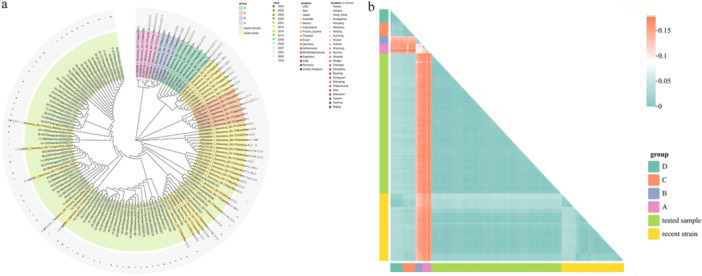
Phylogenetic analysis with 73 filter sequences in our study and globally representative strains from GenBank during 2014–2023. (a) Phylogenetic tree. The maximum likelihood phylogenetic tree was constructed using MEGA X under the GTR + Γ model. Tip labels are colored by variant group (A–D, tested samples, and recent strains from 2021 to 2025). Collection years are indicated by colored dots at the branch tips. Geographic origins are shown on the concentric ring: the inner dots represent countries, and the outer dots represent Chinese cities. Branch support was evaluated using the ultrafast bootstrap method with 10 000 replicates. (b) Heatmap of pairwise nucleotide divergence calculations. Rows and columns are grouped and annotated by variant (A, B, C, D, tested samples, and recent strains from 2021 to 2025).

Phylogenetic analysis based on partial RdRp and VP1 sequences, incorporating 73 filter sequences from our study and globally representative strains from GenBank, showed that the GII.17[P17] strains circulating this season form a distinct clade separate from the ABCD lineage, aligning more closely with the 2021 Romanian outbreak strains (Figure 4a). This phylogenetic relationship was corroborated by nucleotide sequence divergence calculations (Figure 4b). Sequence similarity analysis of our strains showed ≥ 97.8% nucleotide identity to representative GII.17[P17] strains circulating globally from 2021 to 2025, especially with the 2024 Beijing isolate sharing > 99% identity, indicating near‐genetic equivalence. Besides, comparison with strains prevalent during 2021–2023 showed nucleotide divergences ranging from 1% to 2%, while divergence from globally circulating strains during 2024–2025 was generally < 1%, consistent with the expected evolutionary variation within this lineage (Supplementary File 2). No recombination events were detected among strains circulating between 2021 and 2025. Strains identified across multiple cities in China during the 2024–2025 season exhibited high nucleotide sequence conservation, with inter‐regional sequence identity reaching 98.7% or higher. Slightly elevated nucleotide divergence (> 1%) was observed among strains from the early and late phases of the season, primarily in cities spanning from the eastern coastal to central inland regions. These variations remain within the expected range for homologous strains. Temporally, intra‐city nucleotide divergence—such as between strains from Wenzhou in 2024 and 2025—was minimal (≤ 0.0078), indicating sustained local circulation. Geographically, divergence between contemporary Chinese strains and 2023–2025 European/American strains was low (< 0.0078), and substantially lower than divergence from local Chinese strains dating back to 2015 (0.1206–0.1686), supporting the hypothesis of recent intercontinental transmission.

## 4 Discussion

This multicenter surveillance study demonstrates a marked increase in GII.17 norovirus infections among hospitalized children with acute gastroenteritis in China during the 2024–2025 season. GII.17[P17] accounted for 62% of all norovirus‐positive cases, representing a substantial shift from the long‐standing predominance of GII.4 strains. Similar trends have been reported in several European countries and the United States during 2023–2024 [25, 26, 27], suggesting a broader global re‐emergence of this genotype.

Consistent with previous studies, norovirus infections were most frequently detected in young children, particularly those aged 1– < 3 years [38]. This age group showed significantly higher odds of infection in logistic regression analysis. The overall decline in detection rates with increasing age is consistent with the accumulation of protective immunity following repeated exposures during early childhood. Notably, GII.17 norovirus showed a higher incidence than GII.4 across all age groups, indicating strong and broad susceptibility in the pediatric population. Seasonal analysis confirmed a pronounced cold‐weather peak of norovirus activity, with significantly higher odds of infection during winter months in logistic regression analysis. This seasonal pattern aligns with established norovirus epidemiology [37] and reflects favorable environmental and behavioral conditions for viral transmission during colder months.

Geographically, higher odds of norovirus infection were observed in the eastern and central regions of China compared with the northern region, particularly in the eastern/coastal provinces (e.g., Zhejiang and Guangdong) and the south‐western provinces (e.g., Sichuan). Several factors may contribute to these regional differences. Eastern and central regions are characterized by higher population density, greater urbanization, and more frequent inter‐city mobility, which may facilitate rapid dissemination of a dominant lineage such as GII.17[P17]. In contrast, southern regions experience subtropical temperatures and humidity, which may influence viral environmental stability and transmission efficiency. The prevalence of GII.4[P16] virus is slightly higher than GII.17[P17], for example, in southern Dongguan city. Additionally, the detection of relatively rare genotypes, including GI.3[P13] in Dazhou, GII.2[P16] and GII.8[P8] in Ningbo, likely reflects localized introductions or low‐level endemic circulation that is not sustained over time. Ningbo, as a coastal city with strong connectivity, may be more prone to the introduction of diverse norovirus lineages. In contrast, smaller inland cities such as Dazhou, characterized by relatively limited population mobility, may have reduced exposure to a broad range of circulating genotypes, thereby increasing the likelihood of sporadic infections caused by uncommon genotypes. However, regional differences in historical exposure to specific norovirus genotypes may shape population‐level immunity, although this could not be directly assessed in the present study.

Since 2002, norovirus GII.4 has undergone rapid variant turnover and has consistently predominated globally, whereas GII.17 emerged as a prominent lineage only in the mid‐2010s [37]. Early genetic analyses have identified four major GII.17 clusters (A–D), with cluster D becoming dominant since 2013 [36, 39]. The first major variant recognized globally was GII.17 Kawasaki_2014 (GenBank ID: AB983218), identified in Japan during the 2014–2015 winter [16]. In 2021, a genetically distinct GII.17[P17] lineage was linked to a large gastroenteritis outbreak in Romania (Romania_2021, GenBank ID: OP805362) [40] subsequently spread across Europe and North America and accounted for over 80% of GII.17 strains detected during 2023–2024 [25, 26], and included related “C2” subcluster strains isolated in Nizhny Novgorod, Russia (2021–2023) [28]. Phylogenetic analysis indicated that the GII.17[P17] strains circulating in China during the 2024–2025 season are closely related to these global strains, sharing a recent common ancestor (Romania_2021), though accumulating nucleotide divergence suggests incipient subtype emergence. The high nucleotide similarity and low inter‐city genetic divergence observed among Chinese strains (e.g., Wenzhou vs. Kunming) suggest rapid nationwide dissemination of a single dominant lineage rather than multiple independent introductions. The absence of detectable recombination events further supports the genetic stability of the circulating GII.17[P17] strains during this season.

Norovirus evolution is strongly driven by homologous recombination, particularly near the ORF1/ORF2 junction, resulting in extensive diversity of polymerase‐capsid combinations, with at least 14 GI polymerase (GI.P) types and 37 GII.P types described to date [35, 36, 41]. Historically, the GII.17 capsid has been associated with multiple polymerase types, including GII.P13, GII.P4, and GII.P3, until the emergence of the recombinant GII.P17‐GII.17 strain (Kawasaki_2014), which represented a major evolutionary shift [34]. Since then, no further dominant recombinant GII.17 strains have been reported, and both the Romania_2021 lineage and all strains identified in this study belonged to the GII.17[P17] genotype. In contrast, two GII.4 constellations were detected in our dataset: GII.4[P16] and GII.4[P31], accounting for 26% and 1% of norovirus‐positive cases, respectively. GII.4[P31], corresponding to the classical GII.4 Sydney_2012 lineage, was globally predominant between 2009 and 2015. A subsequent recombinant, GII.4 Sydney[P16], emerged around 2015–2016 and since circulated widely [13, 36]. Consistent with this global trend, GII.4[P16] was the predominant GII.4 strain in China during 2024–2025, whereas GII.4[P31] was only detected in three cases in Wenzhou city.

These findings are based on raw counts, more accurately comparing infection risk across cities would require normalization by population size and/or testing volume to account for differences. In addition, all fecal samples were obtained from hospitalized pediatric patients with AGE, since standardized assessments of symptom severity (e.g., mild, moderate, or severe) were not available in clinical diagnosis, norovirus genotype‐specific virulence differences could not be evaluated in this study.

## 5 Conclusion

The epidemiological landscape of norovirus is complex, reflecting its capacity to cause outbreaks across diverse settings and to disproportionately affect vulnerable populations, including young children, the elderly, and immunocompromised individuals. Our study highlights the influence of specific genotype predominance on temporal fluctuations in norovirus infection patterns. The substantial genetic diversity complicates efforts to develop vaccines and continues to challenge disease control. Despite the increasing prevalence of GII.17 virus, the continued detection of GII.4 highlights the co‐circulation of multiple norovirus genotypes. Ongoing surveillance remains essential to monitor emerging variants and to inform public health interventions and vaccine strategies.

## Author Contributions

Xuanyi Wang conceived and designed the study. Wenjing Zheng and Shaoyan Wang performed the experiments. Wenjing Zheng and Xinyue Mu analyzed the data and drafted the manuscript. Tianyi Qiu provided important input on the data analysis and revised the manuscript.

## Conflicts of Interest

The authors declare no conflicts of interest.

## Supporting information


**Supporting Materials File 1:** The GenBank accession numbers for all reference sequences used in this study.

**Supporting Materials File 2:** Detailed nucleotide identity and divergence data for Norovirus GI.17[P17]strains.

## Data Availability

The data that support the findings of this study are available from the corresponding author upon reasonable request.
